# Humoral responses to SARS-CoV-2 mRNA vaccines: Role of past infection

**DOI:** 10.1371/journal.pone.0259703

**Published:** 2021-11-08

**Authors:** Ashley N. Gray, Rachel Martin-Blais, Nicole H. Tobin, Yan Wang, Sarah L. Brooker, Fan Li, Adva Gadoth, Julie Elliott, Emmanuelle Faure-Kumar, Megan Halbrook, Christian Hofmann, Saman Kashani, Clayton Kazan, Otto O. Yang, Jennifer A. Fulcher, Kathie Grovit-Ferbas, Anne W. Rimoin, Grace M. Aldrovandi

**Affiliations:** 1 Division of Pediatric Hematology-Oncology, Department of Pediatrics, David Geffen School of Medicine at University of California, Los Angeles, CA, United States of America; 2 Division of Pediatric Infectious Diseases, Department of Pediatrics, David Geffen School of Medicine at University of California, Los Angeles, CA, United States of America; 3 Division of Infectious Diseases, Department of Medicine, David Geffen School of Medicine at University of California, Los Angeles, CA, United States of America; 4 Jonathan and Karin Fielding School of Public Health, University of California, Los Angeles, California, United States of America; 5 Division of Digestive Diseases, Department of Medicine, David Geffen School of Medicine at University of California, Los Angeles, CA, United States of America; 6 Emergency Medical Services Bureau, Los Angeles County Fire Department (LACoFD), Los Angeles, CA, United States of America; Qatar University, QATAR

## Abstract

Two mRNA vaccines (BNT162b2 and mRNA-1273) against severe acute respiratory syndrome-coronavirus 2 (SARS-CoV-2) are globally authorized as a two-dose regimen. Understanding the magnitude and duration of protective immune responses is vital to curbing the pandemic. We enrolled 461 high-risk health services workers at the University of California, Los Angeles (UCLA) and first responders in the Los Angeles County Fire Department (LACoFD) to assess the humoral responses in previously infected (PI) and infection naïve (NPI) individuals to mRNA-based vaccines (BNT162b2/Pfizer- BioNTech or mRNA-1273/Moderna). A chemiluminescent microparticle immunoassay was used to detect antibodies against SARS-CoV-2 Spike in vaccinees prior to (n = 21) and following each vaccine dose (n = 246 following dose 1 and n = 315 following dose 2), and at days 31–60 (n = 110) and 61–90 (n = 190) following completion of the 2-dose series. Both vaccines induced robust antibody responses in all immunocompetent individuals. Previously infected individuals achieved higher median peak titers (p = 0.002) and had a slower rate of decay (p = 0.047) than infection-naïve individuals. mRNA-1273 vaccinated infection-naïve individuals demonstrated modestly higher titers following each dose (p = 0.005 and p = 0.029, respectively) and slower rates of antibody decay (p = 0.003) than those who received BNT162b2. A subset of previously infected individuals (25%) required both doses in order to reach peak antibody titers. The biologic significance of the differences between previously infected individuals and between the mRNA-1273 and BNT162b2 vaccines remains uncertain, but may have important implications for booster strategies.

## Introduction

The novel coronavirus, severe acute respiratory syndrome-coronavirus 2 (SARS-CoV-2), has swept the globe since December 2019, straining health systems and leading to millions of excess deaths [1]. The development of SARS-CoV-2 vaccines to prevent severe illness and curb transmission is one of the most important public health measures in the fight against this pandemic. In December 2020, two companies, Pfizer-BioNTech and Moderna, were granted emergency use authorizations (EUA) in the United States of America for their mRNA-based SARS-CoV-2 vaccines encoding the spike (S) protein [2,3]. Though the BNT162b2 (Pfizer-BioNTech) and mRNA-1273 (Moderna) vaccines have recently been shown to lead to a robust antibody response following one [4–7] as well as two doses [8], our understanding of the differences in humoral response between these vaccines remains limited. A better understanding of these responses is paramount given limited global vaccine supply, distribution challenges, and the concern for emerging variant SARS-CoV-2 strains that may necessitate the use of additional doses [9,10].

The SARS-CoV-2 RNA genome encodes several immunogenic structural proteins, including spike (S) and nucleocapsid (N). Individuals with natural infection can develop antibodies to both spike and nucleocapsid, whereas infection-naïve, vaccinated persons only produce antibodies directed towards spike [11–13]. These differences in antibody response enable discrimination between prior infection and response to spike glycoprotein-directed vaccines. This study aims to better characterize the antibody responses and initial decay of these responses in individuals after mRNA-based vaccination against SARS-CoV-2 by comparing antibody titers elicited by the two vaccines and between previously SARS-CoV-2-infected and infection-naïve individuals.

## Methods

### Participants

Health services workers at the University of California, Los Angeles (UCLA) and first responders in the Los Angeles County Fire Department (LACoFD) were enrolled on an ongoing basis in a longitudinal cohort study assessing rates of SARS-CoV-2 infection in high-risk individuals beginning in April of 2020 [14]. Health service workers performed observed, self-administered mid-turbinate (MT) nasal swabs for PCR testing every 2 weeks, and provided blood samples for serological analyses every 4 weeks; First responders provided both types of samples every 4 weeks. Participants were defined as immunocompromised if they reported taking medications that cause decreased number or function of lymphocytes on their self-reported study questionnaire. Data from immunocompromised participants data are included and described but not analyzed with the entire cohort as their immunosuppressive medications can affect the ability to produce an adequate humoral response. All participants provided an electronic, signed informed consent prior to participation in the study. No minors were enrolled in the study. All data was de-identified. This research was reviewed and approved by the UCLA Institutional Board Review committee (IRB#20–000478).

### Vaccination and Blood Specimen Collection

Participants received either BNT162b2 or mRNA-1273 through their respective employers based on vaccine availability. Employees of UCLA Health primarily received BNT162b2, while those of LACoFD primarily received mRNA-1273.

We classified participants as either not previously infected (NPI) or previously infected (PI) with SARS-CoV-2 prior to receipt of the first vaccine dose. Participants were categorized as previously infected if they had a positive SARS-CoV-2 RT-PCR from a mid-turbinate or nasopharyngeal swab or had detectable IgG antibody to either the RBD portion of the S protein (anti-Spike) [15] or to the N protein (anti-Nucleocapsid) [16] prior to immunization. Participants were categorized as NPI if they had negative nasal swabs, negative serology and no symptomatic history suggestive of infection prior to their first dose of vaccine. Participants with a history of anosmia and/or ageusia without a positive PCR or antibody test were not classified and therefore not included in any comparisons that required classification.

Participants had blood drawn between 7 days after the first vaccine dose and just prior to the second dose (up to 20 days after the first dose of BNT162b2 and up to 27 days after the first dose of mRNA-1273). Blood was also collected 7–30 days, 31–60 days and 61–90 days after completion of the two-dose series. Not all participants provided blood samples at every time point due to missed appointments or blood draw refusal.

### Antibody measurement and interpretation

Antibody testing was performed using a chemiluminescent microparticle immunoassay to detect antibodies (IgM and IgG) against the RBD domain of the Spike protein, or IgG against Nucleocapsid, per the manufacturer’s instructions and previously published protocols (Abbott Alinity i series, Abbott Laboratories (Chicago, IL) [17,18]. Positive anti-Spike IgM levels are defined as greater than or equal to 1.0 times the index, while negative results were below the limit of detection (LLD) [19]. Positive anti-Spike IgG titers, specifically to the RBD portion of the S protein, were defined as equal to or more than 50 arbitrary units per milliliter (AU/mL), according to the manufacturer’s assay specifications and per the FDA EUA and is represented as the lower limit of quantification (denoted as LLQ). We used the manufacturer provided guidance of anti-Spike IgG at or above 3,950 AU/mL on the assay as a surrogate for high neutralizing antibody titers (95% probability of corresponding to high neutralizing antibody titer at a 1:250 dilution in a plaque-reduction neutralization test (PRNT) assay) [17,18].

### Statistical analysis

Characteristics of participants were compared by type of vaccine received, as well as by previous SARS-CoV-2 infection status. Continuous variables were compared using two-sample t-tests. Categorical variables were compared by Chi-square test (Fisher’s exact test was used if there were fewer than five observations). The medians of anti-Spike IgG between participants previously infected (PI) and not previously infected (NPI) were compared using Wilcoxon rank-sum test or Mann-Whitney U test at four time points: following dose 1, following dose 2, and 31–60 days and 61–90 days after completion of the vaccine series. To evaluate antibody decay, we included participants for whom we had samples from the peak anti-Spike IgG titer window, as well as at least one follow-up at either days 31–60 or 61–90. We excluded immunocompromised participants from statistical analyses of vaccine responses and antibody decay. A longitudinal model with random intercept and slope (RIAS) was used to analyze the natural log-transferred anti-Spike IgG and PI/NPI, adjusted for age, sex, race/ethnicity, occupation and vaccine type. Among NPI, RIAS was used to model the natural log-transferred anti-Spike IgG and vaccine type, adjusted for age, sex, race/ethnicity, and occupation. All statistical analyses were performed utilizing the SAS 9.4 (Cary, NC) and R statistical software (R Core Team, 2014), and p-values <0.05 were considered significant.

## Results

### Participant characteristics

Participant characteristics are described in Table 1. Mean age and age range were similar between PI and NPI groups. Participants receiving the BNT162b2 vaccine were primarily healthcare workers and female (both p<0.0001) and were younger (p = 0.0002) compared to persons receiving the mRNA-1273 vaccine. The majority of participants receiving BNT162b2 self-identified as non-Hispanic ethnicity and white race. No difference in previous infection status between racial/ethnic groups or occupation was observed.

**Table 1:** Characteristics of the participants.

	Mean (SD) or N (%)	No Prior Infection (N = 422)	Prior Infection (N = 38)	BNT162b2/Pfizer (N = 345)	mRNA-1273/Moderna (N = 116)
		NS*		**p = 0.0002** *	
**Age**	42.3 (11), range 20–71	42.1 (11), range 20–71	44.1 (11.3), range 29–70	41.2 (11), range 23–71	45.5 (10.3), range 20–66
**Gender**		**NS** ^**^**^		**p = <0.0001** ^**^**^	
Female	287 (62.3)	268 (63.5)	19 (50)	246 (71.3)	41 (35.3)
Male	173 (37.5)	153 (36.3)	19 (50)	98 (28.4)	75 (64.7)
Prefer not to say	1 (0.2)	1 (0.2)		1 (0.3)	
**Race/Ethnicity**		**p <0.0001** ^#^		**p = 0.0002** ^^^	
Hispanic	63 (13.7)	55 (13)	8 (21.1)	36 (10.4)	27 (23.3)
Non-Hispanic Asian	98 (21.3)	91 (21.6)	7 (18.4)	84 (24.4)	14 (12.1)
Non-Hispanic Black	21 (4.6)	17 (4)	4 (10.5)	12 (3.5)	9 (7.8)
Non-Hispanic Other	34 (7.4)	31 (7.4)	3 (7.9)	29 (8.4)	5 (4.3)
Non-Hispanic White	218 (47.3)	203 (48.1)	14 (36.8)	167 (48.4)	51 (44)
Unknown	27 (5.9)	25 (5.9)	2 (5.3)	17 (4.9)	10 (8.6)
**Occupation**		**p = 0.007** ^**^**^		**p = <0.0001** ^#^	
Firefighters	84 (18.2)	70 (16.6)	13 (34.2)	2 (0.6)	82 (70.7)
Healthcare Workers	377 (81.8)	352 (83.4)	25 (65.8)	343 (99.4)	34 (29.3)
**Vaccine Type**		**p = 0.0009** ^^^			
BNT162b2/Pfizer-BioNTech	345 (74.8)	325 (77)	20 (52.6)		
mRNA-1273/Moderna	116 (25.2)	97 (23)	18 (47.4)		
**History of Prior Infection**				**p = 0.0009** ^^^	
Unknown	1 (0.2)				1 (0.9)
No prior Infection	422 (91.5)			325 (94.2)	97 (83.6)
Have prior infection	38 (8.2)			20 (5.8)	18 (15.5)

NS = Not Significant. P-value calculated by

*T-test,

^^^Chi-square test,

^#^Fishers exact test.

### Anti-Spike IgG responses following vaccination

**Fig 1:**
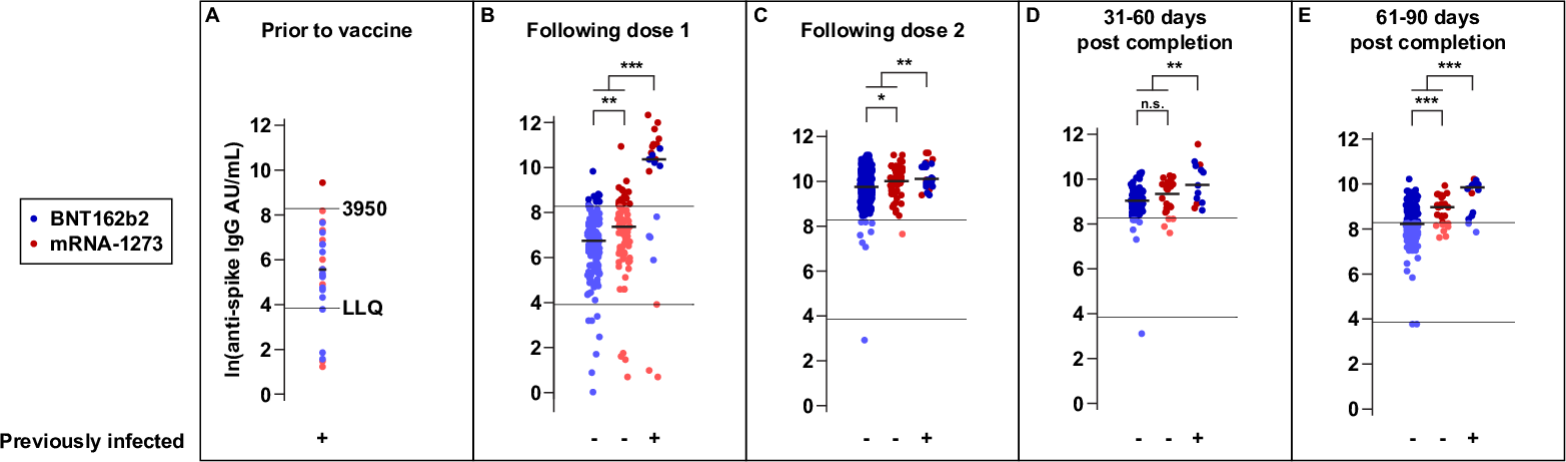
Longitudinal immune response to anti-Spike IgG following each vaccination dose and up to 90 days post-vaccination. Anti-Spike IgG from prior to vaccination to 90 days after completion of the vaccine schedule for participants who received BNT162b2 (blue) and mRNA-1273 (red) by prior infection status (+/-) demonstrate higher levels of anti-Spike IgG in participants with prior infection. **Panel A** shows participants with prior infection and their anti-Spike IgG level prior to vaccination. Panels B-E show participants with and without prior infection by vaccine type. Participants with prior infection were combined regardless of vaccine type. Anti-Spike IgG levels are shown following the 1^st^ dose (**Panel B**), following the 2^nd^ dose (**Panel C**), and 31–60 days (**Panel D**) and 61–90 days after completion (**Panel E**) of the 2-dose series. Positive anti-Spike IgG titers were defined as at or above the lower limit of quantification (LLQ). High level anti-Spike IgG responses were defined at or above 3,950 AU/mL. Horizontal black lines indicate median titer level within each group. P-values compare anti-Spike IgG titers for participants with prior infection versus no prior infection and for participants with no prior infection by vaccine type, * p < 0.05 and ** p < 0.01, ***p <0.001, n.s. = not significant. There were two participants on immunosuppressive medications who did not respond to vaccination (the blue dots at or below the LLQ in panels C, D, and E). Their data is shown but not included in the analyses.

Prior to vaccination, most 20/25 (80%) PI participants (PIs) had detectable anti-Spike IgG titers (median 260 AU/mL), with only one displaying a level greater than 3,950 AU/mL (Figs 1A and S1), a threshold associated *in vitro* with high neutralizing antibody activity [17,18]. Following the first dose of either vaccine, 91% (20/22) of PIs participants had positive anti-Spike IgG titers, and 68% (15/22) had levels 3,950 AU/mL including two participants who reported taking immunosuppressive medications (Fig 1B).

Fifteen PIs were both immunocompetent and more than 21 days beyond natural infection at the time of the first vaccination dose. Of these, 5 did not achieve antibody levels ≥3950 AU/mL. We compared these 5 PIs (<3950 AU/mL) to 10 PIs (≥3950 AU/mL) and found no significant differences in age, sex, race, symptom history, history of febrile illness, comorbidities or time to SARS-CoV-2 infection. Characteristics of these 5 PIs were reviewed in S1 Table. S3 Fig displays the anti-Spike IgG titers following the first dose of the vaccine by days between SARS-CoV-2 infection and first dose.

In contrast, 95% (218/229) of NPIs had responses above the LLQ, but only 12% (28/229) with levels 3,950 AU/ml following the first vaccine dose. PIs had higher median titer responses than NPIs (median 32,727 AU/mL versus 1,585 AU/mL NPI/mRNA-1273 and 869 AU/mL NPI/BNT162b2, p = 0.002; Figs 1B and S1). Following the second dose, all PIs had antibody levels 3,950 AU/ml and 288/297 (97%) of NPIs also reached this threshold (Fig 1C). Of note, peak antibody titers in NPIs were significantly associated with age (p = 0.012) but not sex, with younger participants achieving higher anti-Spike IgG levels (S2 Fig). Anti-Spike IgG levels remained significantly higher in PI versus NPI at 31–60 (p = 0.003) and 61–90 days (p<0.001) post-completion (Fig 1D and 1E).

**Fig 2:**
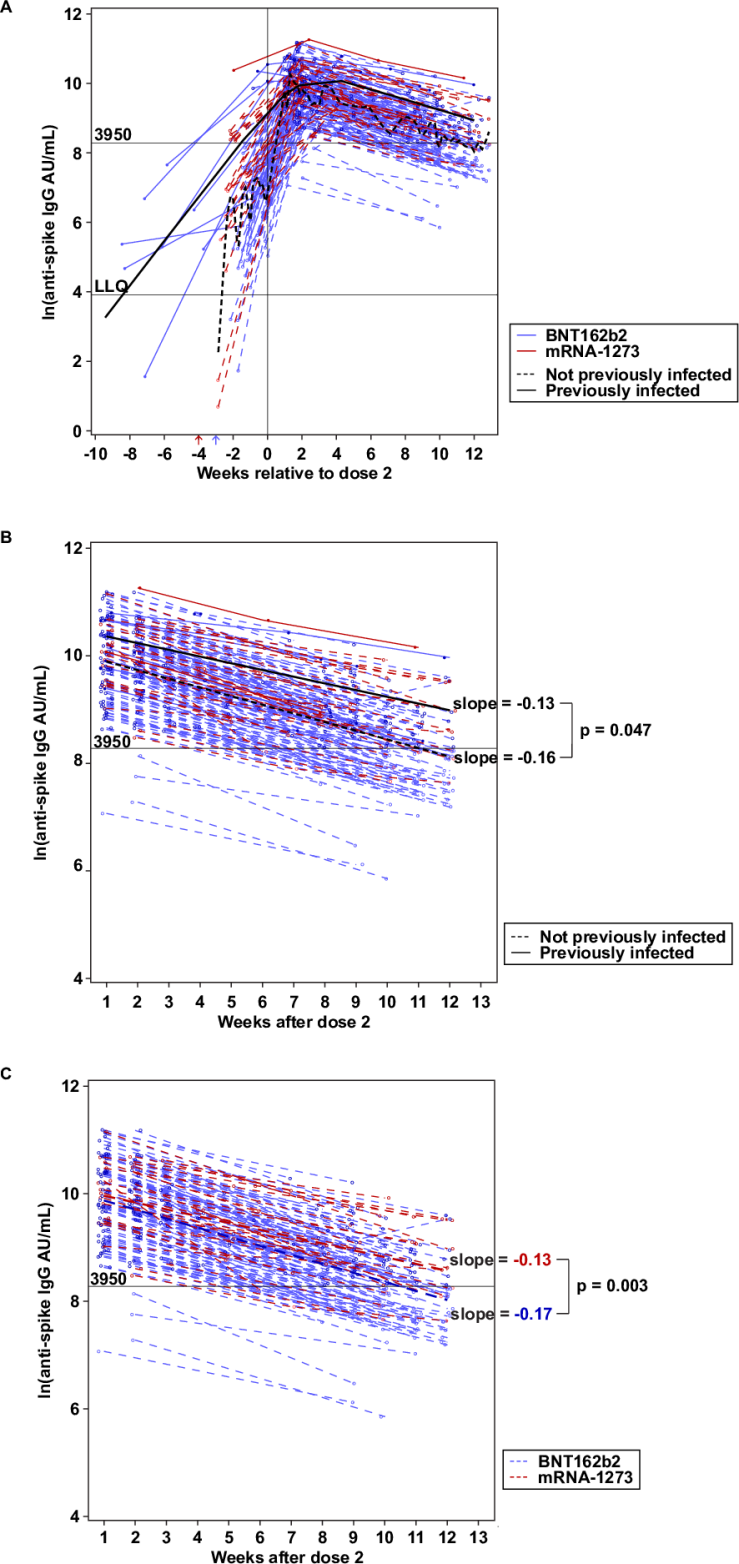
Anti-Spike IgG antibody dynamics and decay up to 90 days post-vaccination. Anti-Spike IgG dynamics and decay in previously infected (solid line, filled circles) versus not previously infected (dashed line, open circles) participants by vaccine type (BNT162b2 in blue, mRNA-1273 in red), as well as slower rate of antibody decay in previously infected individuals (Panel B) and in not previously infected individuals following vaccination with mRNA-1273. **Panel A** demonstrates the more rapid rise of antibody titers in PI Individuals following the first dose. Anti-Spike IgG titer is displayed by time before and after the second vaccine dose (vertical black line). Blue and red arrows mark the approximate timing of the 1^st^ vaccine dose for BNT162b2 and mRNA-1273, respectively. **Panel B and C** shows the anti-Spike IgG decay after the peak is reached following the 2^nd^ vaccine dose. The rate of decay is slower in PI (N = 12) versus NPI (N = 168) (Panel B) and in recipients of mRNA-1273 (N = 30) versus BNT162b2 (N = 138) (Panel C).

The dynamics of the antibody responses and decay are shown in Fig 2. Anti-Spike IgG levels rose rapidly in response to the first vaccination for most PIs (solid line), whereas NPIs showed a steeper rise after the second dose of the vaccine (dashed line) (Fig 2A). The rate of antibody decay was faster in NPIs versus PIs, at -0.16 versus -0.13 ln-units/week; p = 0.047 (Fig 2B). This appeared to be mostly driven by recipients of BNT162b2, the majority of our subjects serially sampled following dose 2.

### Comparison of Anti-Spike IgG responses by vaccine received

Antibody levels for NPIs were significantly higher in recipients of the mRNA-1273 compared to the BNT162b2 vaccine at all timepoints except 30–60 days after dose 2 (Table 2). Notably, the differences in antibody levels between mRNA-1273 and BNT162b2 were approximately 2-fold or less.

**Table 2:** Summary statistics of anti-spike IgG responses by vaccine type and infection history.

Vaccine Dose	Infection Status/Vaccine Type	N	Median AU/mL (range)	p-value^a^	p-value^b^
**Before vaccine**	PI/mRNA-1273	8	1224 (50–12800)		n.s.
	PI/BNT162b2	15	198 (50–2110)		
**Following Dose 1**	PI/mRNA-1273	11	61608 (50–229362)	0.0002	
	PI/BNT162b2	9	23450 (368–49758)		
	NPI/mRNA-1273	76	1585 (50–56042)		0.005
	NPI/BNT162b2	150	869 (50–19013)		
**Following Dose 2**	PI/mRNA-1273	8	47569 (11970–79441)	0.0017	
	PI/BNT162b2	14	24078 (11945–48940)		
	NPI/mRNA-1273	56	22473 (4803–70819)		0.0289
	NPI/BNT162b2	237	17275 (1174–72155)		
**After 31–60 days**	PI/mRNA-1273	3	42839 (7628–104805)	0.0027	
	PI/BNT162b2	9	17024 (5485–47778)		
	NPI/mRNA-1273	24	11662 (1996–25267)		n.s.
	NPI/BNT162b2	74	8518 (1525–29336)		
**After 61–90 days**	PI/mRNA-1273	5	21563 (14799–27428)	< .0001	
	PI/BNT162b2	11	16928 (2737–26076)		
	NPI/mRNA-1273	26	7873 (2076–20335)		0.0009
	NPI/BNT162b2	148	3738 (349–27024)		

^a^ Wilcoxon rank-sum test comparing medians between previously infected (PI) versus not previously infected (NPI) participants.

^b^ Wilcoxon rank-sum test comparing medians between NPI receiving mRNA-1273 versus NPI receiving BNT162b2.

As expected, levels of circulating antibodies decreased 61–90 days post-completion of their immunization series (S4 Fig). The BNT162b2 group displayed a more rapid antibody decay following the second vaccine dose when compared to the mRNA-1273 group (-0.17 versus 0.13 Ln-units/week, p = 0.003) (Fig 2C). While 32% (7/22) of those receiving mRNA-1273 had titers <3,950 AU/mL by 61–90 days following dose 2, more than half (52%, 84/163) of those receiving the BNT162b2 vaccine had titers <3,950 AU/mL (median = 3775 AU/mL) (Fig 1D and 1E).

### Spike glycoprotein IgM levels by previous infection status

Anti-Spike IgM levels were assessed prior to vaccination, and following both vaccine doses (S5 Fig). Anti-Spike IgM responses were positively correlated with anti-Spike IgG titers following dose 1 and dose 2 (r^2^ = 0.67 and 0.44 respectively). Anti-Spike IgM responses were not associated with PI/NPI or vaccine type following either dose. The percentage of participants with a positive anti-Spike IgM titer following dose 1 was 51% (73/143) and following dose 2 was 53% (158/297), and 10% (34/335) were detectable at both time points.

## Discussion

The rapid development and deployment of mRNA-based vaccines against SARS-CoV-2 spike protein has provided effective tools to combat a pandemic that has claimed over 3 million lives to date. Given the current limited vaccine supply and the rush to vaccinate as many individuals as possible, vaccination efficiency is necessary. One method of vaccine conservation being considered is the use of single doses or extended-interval dosing for individuals who have baseline antibody responses to SARS-CoV-2 due to previous infection. To examine this possibility, we compared the humoral response to SARS-CoV-2 spike protein following the 2-dose regimen of BNT162b2/Pfizer-BioNTech or mRNA-1273/Moderna vaccine up to 90 days after vaccination in previously infected as compared with infection-naïve individuals.

Our data demonstrated a robust anti-Spike IgG response in most individuals with a prior SARS-CoV-2 infection following the first dose of either mRNA-based vaccine, reflective of a robust memory response and consistent with other recent reports [4–7]. Strikingly, we found that 25% of previously-infected individuals did not reach titers of 3,950 AU/mL following one vaccine dose and required the second dose to cross this threshold. None were on immunosuppressive medications. This suggests that a significant portion of individuals who are predicted to have a robust response after 1 dose are left more vulnerable to infection than expected. For the remaining 68% of PIs with robust responses, our data confirm the elevated vaccine-induced anti-Spike IgG responses in individuals with prior infections that have been seen in other studies [4–7]. Additionally, in infection-naïves, antibody titers induced by mRNA-1273 were approximately 2-fold higher than those induced by BNT162b2, the biological significance of which is unknown. Given the limited number of PIs in this study, a larger sample size and regular interval testing will be needed to better characterize the difference seen in our data. Finally, whereas infection-naïve had a significant increase in anti-Spike IgG level following a second dose of vaccine regardless of vaccine type, previously infected did not show a significant increase after the second dose. This suggests that there may be an immunologic ceiling after which additional antigenic stimulation does not lead to an increase in antibody levels.

Data from this study demonstrate that the anti-Spike IgG response following the first vaccine dose may display greater individual variation than that following the second dose. We identified a small number of participants who had anti-Spike IgG antibody levels below the LLQ. This group of “delayed responders” included participants in both previously infected and infection-naïve groups which indicated that there may be a subset of individuals who do not respond to the first vaccine dose as expected regardless of their infection status. Most of these participants were infection-naïve, and the lack of response was independent of vaccine type. “Delayed responders” to both mRNA-1273 and BNT162b2 were also identified by Krammer *et al*. when followed longitudinally [6]. Further investigations into these “delayed responders” may be helpful to identify individuals who are at greatest risk for breakthrough infections. Nearly all of these “delayed responders” had a robust increase in antibody titer following the second dose, which supports maintaining the 2-dose regimen.

The durability of protection from severe disease following a previous infection and vaccination has yet to be determined. We demonstrate that antibody titers decrease by approximately half between 61–90 days after the second dose of vaccine. This decay is expected in the absence of ongoing antigenic stimulation, and is consistent with the half-life of anti-Spike IgG post-natural infection, which has been shown to be 36–52 days [8,15]. However, we found the rate of decay is faster in vaccinated infection-naïve individuals and also faster in recipients of BNT162b2 than recipients of mRNA-1273. A larger sample size and longer longitudinal follow-up will be needed to determine the significance of this observation.

An anti-Spike IgM response was observed in 73% after either the first or second vaccine dose. Only 10% had IgM responses detected at both time points. This is consistent with studies showing an inconsistent anti-Spike IgM response prior to production of IgG in both asymptomatic carriers and hospitalized patients [20]. These data suggest that anti-Spike IgM may not be a reliable predictor of immune response to either infection or vaccination.

Strengths of our study include large numbers of well-characterized participants in a prospective, longitudinal cohort receiving either mRNA-1273 or BNT162b2 through their employers as well as the use of an FDA EUA approved assay to quantitate antibody responses. Limitations include the variable time of sampling after vaccine doses, which may not reflect each individual’s full response to each dose. Also, the previously infected individuals had natural infections over a broad timeframe prior to vaccination which may affect the antibody kinetics post-vaccination. Additionally, the sample size of previously infected individuals in our cohort was small as was the number of individuals who provided samples more than 2 months following immunization with mRNA-1273. Our participants were relatively young so their responses may not reflect responses in the elderly. Finally, there was potentially more exposure to SARS-CoV-2 post-vaccination in our participants from LACoFD, the majority of whom received mRNA-1273, so if ongoing exposure leads to natural boosting of antibody responses post-vaccination, this may confound the differences seen between mRNA-1273 and BMT162b2. Larger longitudinal studies with regular interval testing will be helpful in further characterizing the differences in peak antibody titers with respect to age, mRNA vaccine type, and in decay rates both in previously infected and infection-naïve persons. Continued studies of antibody responses to emerging SARS-CoV-2 variants types and neutralizing antibody assays to better define protection following vaccination will be continually needed [8]. As an example, on August 16, 2021, the CDC is recommended a 3^rd^ vaccine dose to boost the immune response and durability in the immunocompromised population [21]. The true durability of protection from both infection and vaccination requires characterization of all adaptive immune responses, circulating and long-lived memory B-cell and T-cell (CD4 and CD8) responses, as antibody titers and decay reflect only one aspect of immune protection. There is mounting evidence that robust T cell responses and antibody titer durability are related to history of previous infection and disease severity [22,23]. Investigations into thses responses over time will help determine vaccination strategies in the future.

In conclusion, this study adds to the growing body of evidence that single doses of either mRNA-based vaccine provide a significant anti-Spike IgG response in most previously infected individuals. However, individuals with a slow delayed response following the first vaccination, regardless of their infection history, may require the second vaccine dose to reach antibody levels necessary for disease prevention. We also found that individuals without prior infection reach high anti-Spike IgG levels but experience a faster rate of decay of their antibodies than previously infected individuals. Lastly, the biologic significance of the differences between previously infected individuals and between the mRNA-1273 and BNT162b2 vaccines remains uncertain but may have important implications for booster strategies.

## Supporting information

S1 Fig Timing of anti Spike IgG sampling following each vaccination dose and up to 90 days post-vaccination. Scatter plot of each participant’s anti-Spike IgG titer from prior to vaccination to 90 days after completion of the vaccine schedule by weeks relative to the event. Participants are displayed by vaccine received BNT162b2 (blue) and mRNA-1273 (red) and by prior infection status (previously infected (filled circles) and not previously infected (open circles)). Positive anti-Spike IgG titers were defined as at or above the lower limit of quantification (LLQ). High level anti-Spike IgG by responses were defined at or above 3,950 AU/mL.
(PDF)

S2 Fig Post-vaccination anti-Spike IgG antibody levels by age. Scatter plot displaying each NPI participant’s anti-Spike IgG titers post-second vaccination as a function of age. Black dashed line indicates the regression line by age, -0.013 (p<0.009). High level anti-Spike IgG by responses were defined at or above 3950 AU/mL.
(PDF)

S3 Fig Time since infection and anti-Spike IgG response after dose 1 in previously infected persons. Scatter plot displaying participants with prior infection and their anti-Spike IgG titers after the 1^st^ vaccine dose (BNT162b2 in blue and mRNA-1273 in red) as a function of time since prior infection. The solid black line indicates the regression line by days after natural infection.
(PDF)

S4 Fig Antibody levels over time in participants with no prior infection following dose 2 of the vaccine. Scatter plot displaying participants with no prior infection and their anti-Spike IgG titers after the 2^nd^ vaccine dose (BNT162b2 in blue and mRNA-1273 in red) as a function of time.
(PDF)

S5 Fig Anti-Spike IgM levels in previously infected versus not previously infected participants. Scatter plot displaying the anti-Spike IgM level prior to vaccination, following dose 1, and following dose 2 out to 80 days. Participants who received BNT162b2 (blue) and mRNA-1273 (red) were separated by prior infection status (previously infected (filled circles) and not previously infected (open circles)). Anti-Spike IgM titers are measured via chemiluminescence immunoassay which is expressed as log of AU (arbitrary units). Positive anti-Spike IgM titers were defined as at or above the lower limit of detection denoted as LLD (horizontal solid black line).
(PDF)

S1 Table Characteristics of the five previously infected participants with an anti-Spike IgG <3,950AU/mL after their first vaccine dose. (DOCX)
