# Supplementary figures and images for: Humoral responses to SARS-CoV-2 mRNA vaccines: Role of past infection
Source: PLoS One. 2021 Nov 8;16(11):e0259703. doi: 10.1371/journal.pone.0259703 (PMC8575273; doi:10.1371/journal.pone.0259703)

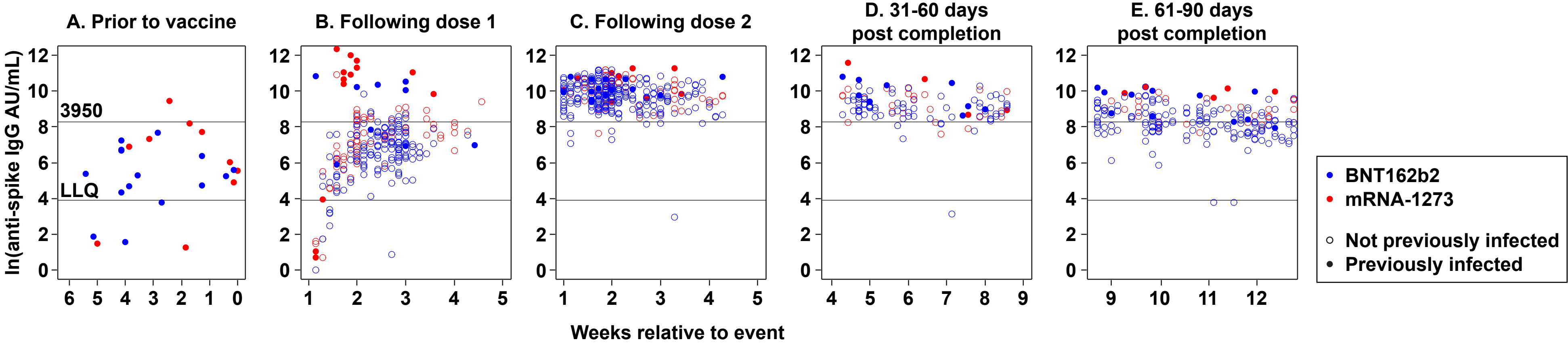

Supplement: S1 Fig — Scatter plot of each participant’s anti-Spike IgG titer from prior to vaccination to 90 days after completion of the vaccine schedule by weeks relative to the event. Participants are displayed by vaccine received BNT162b2 (blue) and mRNA-1273 (red) and by prior infection status (previously infected (filled circles) and not previously infected (open circles)). Positive anti-Spike IgG titers were defined as at or above the lower limit of quantification (LLQ). High level anti-Spike IgG by responses were defined at or above 3,950 AU/mL. (PDF) [file pone.0259703.s001.pdf]

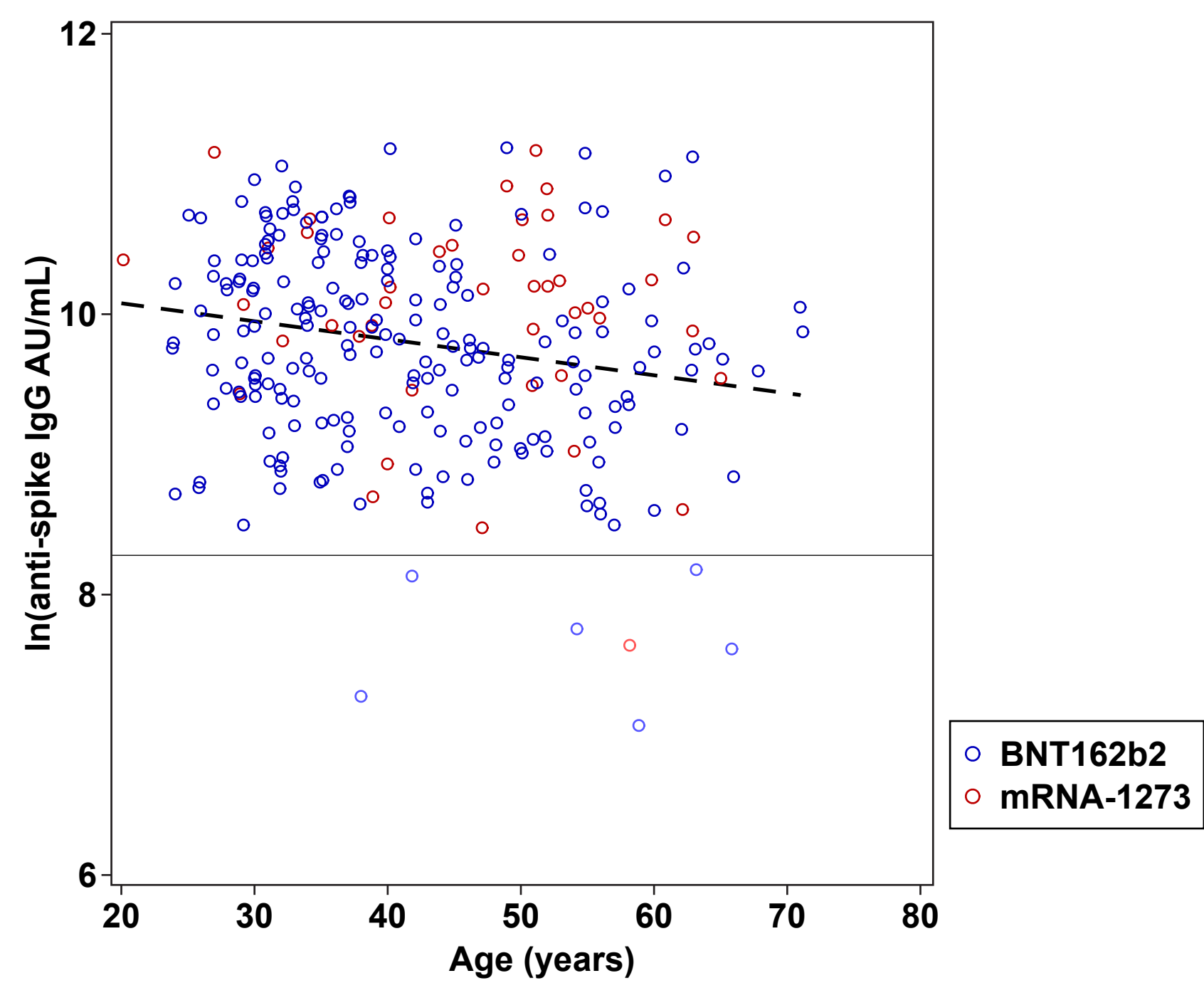

Supplement: S2 Fig — Scatter plot displaying each NPI participant’s anti-Spike IgG titers post-second vaccination as a function of age. Black dashed line indicates the regression line by age, -0.013 (p<0.009). High level anti-Spike IgG by responses were defined at or above 3950 AU/mL. (PDF) [file pone.0259703.s002.pdf]

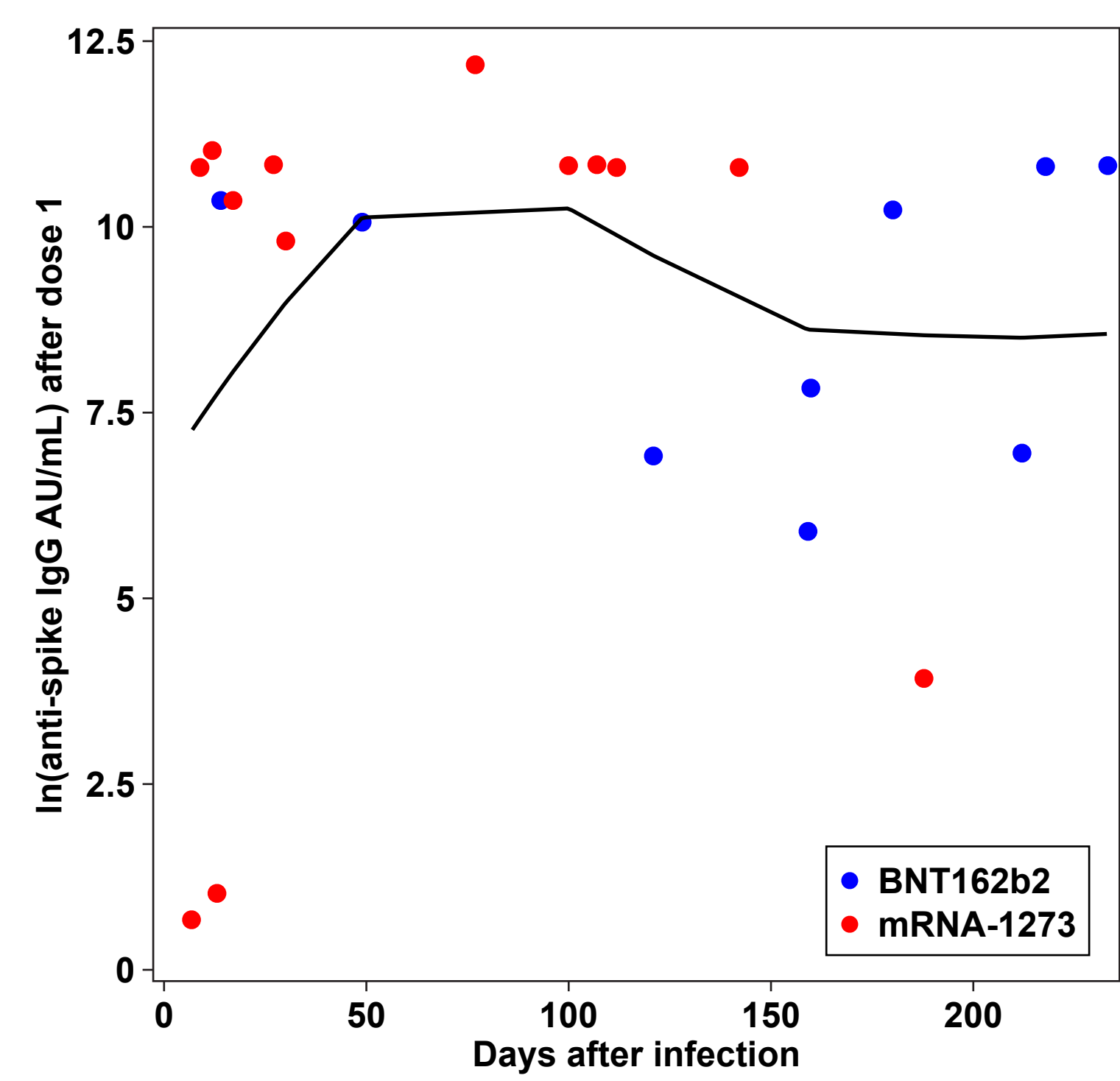

Supplement: S3 Fig — Scatter plot displaying participants with prior infection and their anti-Spike IgG titers after the 1st vaccine dose (BNT162b2 in blue and mRNA-1273 in red) as a function of time since prior infection. The solid black line indicates the regression line by days after natural infection. (PDF) [file pone.0259703.s003.pdf]

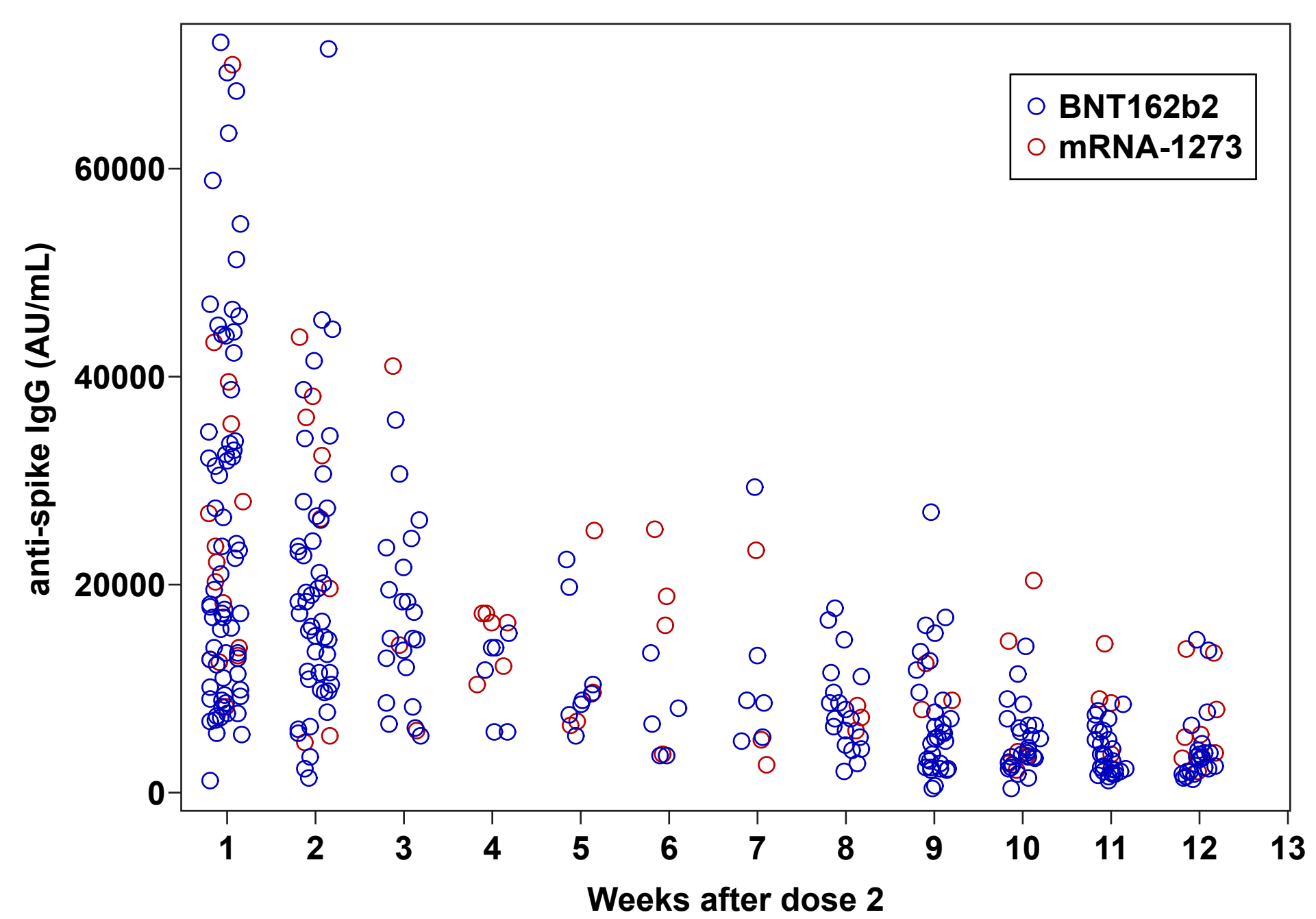

Supplement: S4 Fig — Scatter plot displaying participants with no prior infection and their anti-Spike IgG titers after the 2nd vaccine dose (BNT162b2 in blue and mRNA-1273 in red) as a function of time. (PDF) [file pone.0259703.s004.pdf]

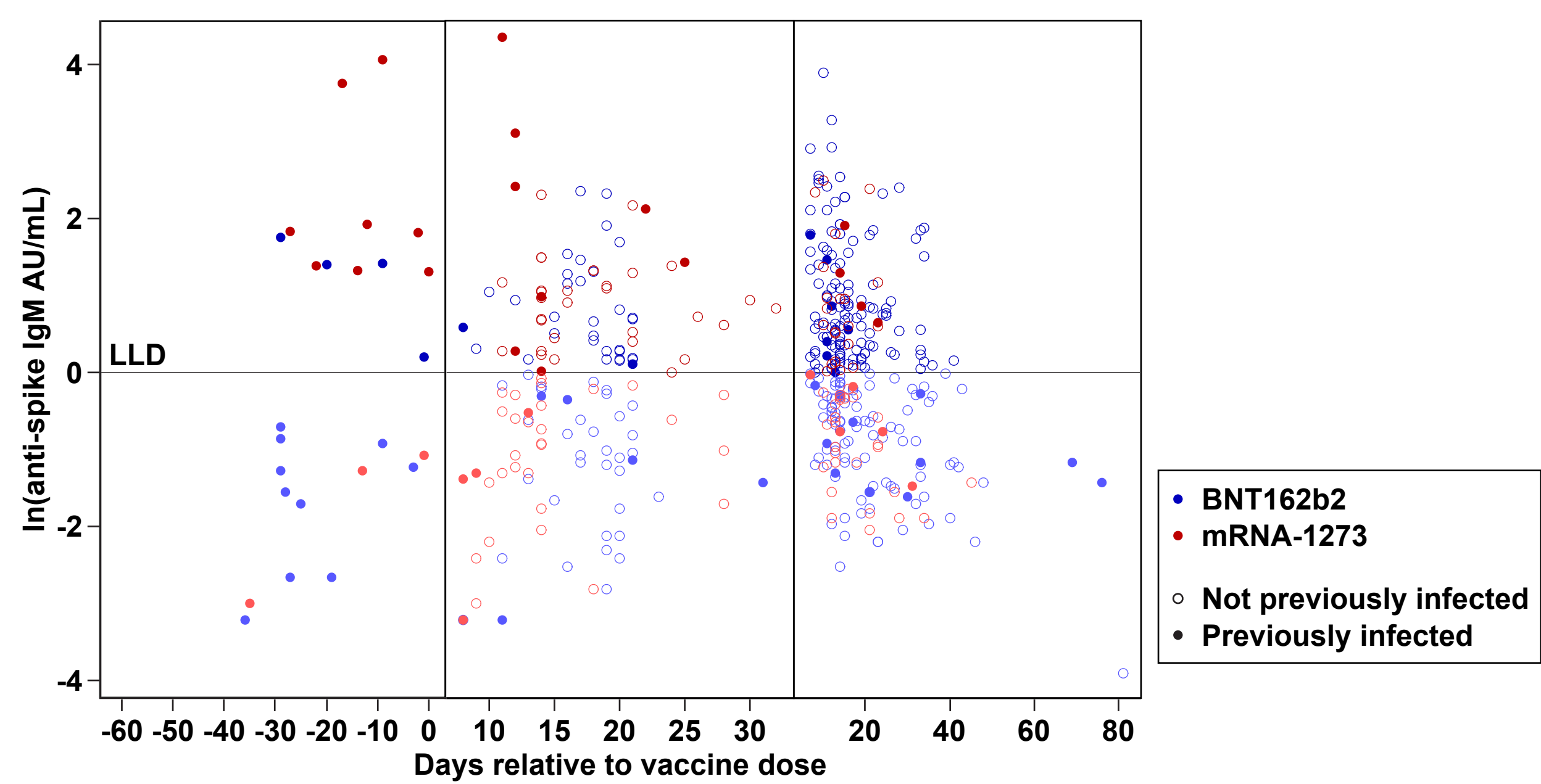

Supplement: S5 Fig — Scatter plot displaying the anti-Spike IgM level prior to vaccination, following dose 1, and following dose 2 out to 80 days. Participants who received BNT162b2 (blue) and mRNA-1273 (red) were separated by prior infection status (previously infected (filled circles) and not previously infected (open circles)). Anti-Spike IgM titers are measured via chemiluminescence immunoassay which is expressed as log of AU (arbitrary units). Positive anti-Spike IgM titers were defined as at or above the lower limit of detection denoted as LLD (horizontal solid black line). (PDF) [file pone.0259703.s005.pdf]
