# Supplementary material for: Humoral responses to SARS-CoV-2 mRNA vaccines: Role of past infection
Source: PLoS One. 2021 Nov 8;16(11):e0259703. doi: 10.1371/journal.pone.0259703 (PMC8575273; doi:10.1371/journal.pone.0259703)
Supplement: S1 Table — (DOCX) [file pone.0259703.s006.docx]

**Supplemental Table 1:** Characteristics of the Five Previously Infected Participants with an Anti-Spike IgG <3,950AU/mL after their First Vaccine Dose

| Subject | Age (years) | Sex | Method of Determining Prior Infection | Vaccine Type | Interval between infection and vaccination | Pre-vaccine Anti-Spike IgG (AU/mL) | Days After Dose 1 | Post-Dose 1 Anti-Spike IgG (AU/mL) | Days After Dose 2 | Post-Dose 2 Anti-Spike IgG (AU/mL) | Medical History/  Medications | Illness Severity* |
| --- | --- | --- | --- | --- | --- | --- | --- | --- | --- | --- | --- | --- |
| 1 | 52 | M | Anti-Nucleocapsid IgG positive | mRNA-1273 | >6 months | <50 | 9 | 50.8 |  | NA | Hypertension/Unknown | Asymptomatic |
| 2 | 51 | M | Anti-Spike IgG positive | BNT162b2 | >5 months | 216.6 | 11 | 367.6 | 11 | 11944.9 | Hypertension/Atorvastatin, amlodipine, losartan, bupropion | Asymptomatic |
| 3 | 66 | F | Anti-RBD IgG positive | BNT162b2 | >6 months | 107.3 | 31 | 1047.6 | 13 | 17435.6 | Hypertension/Benazepril, amiodarone | Asymptomatic |
| 4 | 46 | M | Outside PCR positive | BNT162b2 | 4 months | <50 | 21 | 1004.9 | 11 | 21254.5 | Diabetes/Insulin via pump, lisinopril, lovastatin | Asymptomatic |
| 5 | 30 | F | Anti-RBD IgG positive | BNT162b2 | >5 months | <50 | 16 | 2520.4 | 99 | 2859.3 | Asthma/None | Moderate |

*Participant self-reported symptoms
